# Phytosterols and Cardiovascular Disease

**DOI:** 10.1007/s11883-021-00964-x

**Published:** 2021-09-01

**Authors:** Umidakhon Makhmudova, P. Christian Schulze, Dieter Lütjohann, Oliver Weingärtner

**Affiliations:** 1 Klinik Für Innere Medizin I, Universitätsklinikum Jena, Am Klinikum 1, 07747 Jena, Germany; 2 Institut für klinische Chemie und klinische Pharmakologie, Universitätsklinikum Bonn, Bonn, Germany

**Keywords:** Cholesterol metabolism, Phytosterols, ABCG5/G8, NPC1L1, Atherosclerosis

## Abstract

**Purpose of Review:**

Coronary heart disease is the leading cause of mortality worldwide. Elevated blood cholesterol levels are not only the major but also the best modifiable cardiovascular risk factor. Lifestyle modifications which include a healthy diet are the cornerstone of lipid-lowering therapy. So-called functional foods supplemented with plant sterols lower blood cholesterol levels by about 10–15%.

**Recent Findings:**

In the recent revision of the ESC/EAS dyslipidemia guideline 2019, plant sterols are recommended for the first time as an adjunct to lifestyle modification to lower blood cholesterol levels. However, the German Cardiac Society (DGK) is more critical of food supplementation with plant sterols and calls for randomized controlled trials investigating hard cardiovascular outcomes. An increasing body of evidence suggests that plant sterols per se are atherogenic.

**Summary:**

This review discusses this controversy based on findings from in vitro and in vivo studies, clinical trials, and genetic evidence.

## Introduction

According to the World Health Organization (WHO, 2017), 17.9 million humans die each year from cardiovascular diseases, which accounts for a third of all deaths worldwide [1]. The most common manifestation among cardiovascular diseases is coronary heart disease (CHD). Low-density lipoprotein (LDL-C) levels correlate with coronary heart disease (CHD) [2]. LDL-C reduction decreases the risk for CHD and major coronary events [3]. Therefore, LDL-C reduction is the central goal of the recently revised ESC/EAS guidelines for the management of dyslipidemias: lipid modification to reduce cardiovascular risk and many other international guidelines such as the National Lipid Association (NLA) [4], USA, the National Institute for Health and Care Excellence (NICE) [5], and the American College of Cardiology/American Heart Association (ACC/AHA) [6] to name only a few. Of note, the ESC/EAS dyslipidemia guidelines included in the revised version of 2019 for the first time that plant sterols (phytosterols) are a recommended therapeutic strategy to lower blood cholesterol levels. The ESC/EAS joint guidelines recommend 2 g phytosterol supplementation in individuals with high cholesterol levels at intermediate and low cardiovascular risk; in those who do not qualify for pharmacotherapy, in high- and very-high-risk patients on top of pharmacotherapy who fail to achieve LDL-C-goals or who cannot be treated with statins, and in individuals with familial hypercholesterolemia [7••]. The American Heart Association, on the other hand, restricts the use of phytosterols to patients with familial hypercholesterolemia and for secondary prevention and emphasizes that more information is required to recommend plant sterols in the general population [6]. The National Institute for Health and Care (NICE) in the United Kingdom recommends that anyone with increased risk of CHD not use plant sterols or plant stanols as part of their cholesterol-lowering strategy [8]. Likewise, the German Federal Institute for Risk Assessment (BfR) does not recommend the use of plant sterol-enriched foods and pushes that no more market approval should be awarded to phytosterol-enriched functional foods [9]. Finally, the Germany Cardiac Society (DGK) calls for randomized, controlled trials investigating hard cardiovascular outcomes for foods that are supplemented with plant sterols and criticizes the recently revised ESC/EAS recommendation [10•]. This review summarizes the current evidence on plant sterols and cardiovascular risk.

## Plant Sterol Biochemistry and Physiology

Both cholesterol and phytosterols belong to the family of triterpenes. They have a tetracyclic ring and carbon-linked side chain. Plant sterols differ from cholesterol by structural modification within the side chain in position C24. Plant stanols are saturated sterols with a double-bind at the C5-atom in the B-ring [11]. The most common plant sterols in the diet are campesterol and β-sitosterol (they constitute about 65% and 30%, respectively) and the most common stanols are 5α-saturated stanols (generally plants have very little amounts of stanols) [11].

**Fig. 1:**
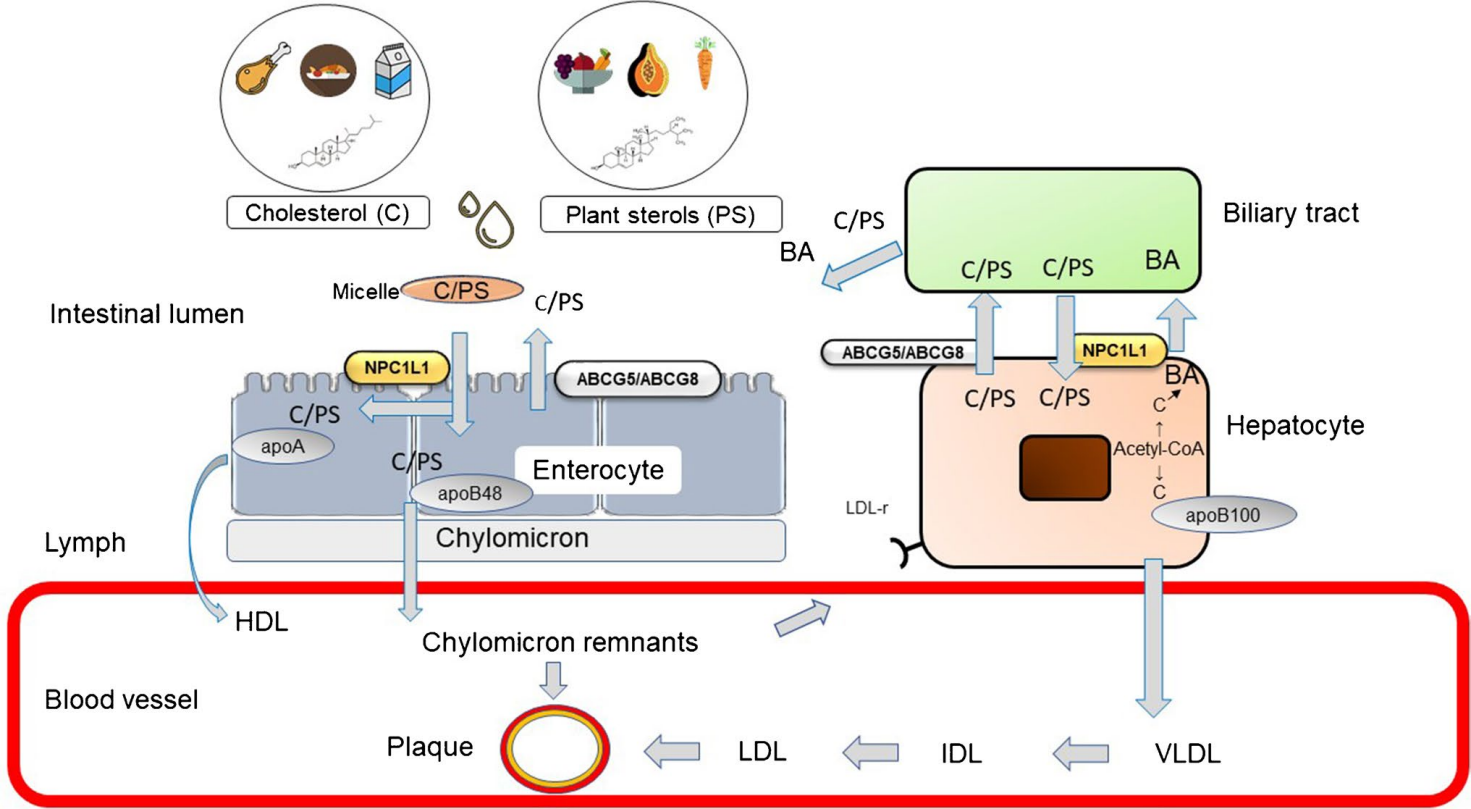
Cholesterol and plant sterol metabolism. Cholesterol (C) and plant sterols (PS) from the diet are incorporated into micelles in the small intestine and are transported to the enterocyte mucosa via the sterol transporter Niemann-Pick C1-like 1 protein (NPC1L1). Free PS and “excess” C are secreted back into the lumen by the ABCG5/ABCG8 heterodimer tandem transporter. Esterified cholesterol and plant sterols are transported in chylomicrons, secreted into the lymph, converted to chylomicron remnants, and taken up in the liver, whereas small amounts of free C/PS leave the enterocyte via apoA-containing HDL. Chylomicron remnants also contribute to atherosclerotic plaque formation in the arterial wall. Thus, the hepatic pool of sterols consists of endogenously synthesized C and dietary C/PS. Also in the hepatocyte, NPC1L1 absorbs sterols and ABCG5/8 secretes them into the bile. Part of cholesterol is converted into bile acids (BA) which are transported in free and conjugated form by specific BA transporters into the bile. In the liver, very low-density cholesterol (VLDL) is formed from C, lipoproteins (containing mostly apoB100), and tryglycerides. In the bloodstream, VLDL is converted to intermediate-density cholesterol (IDL) and low-density cholesterol (LDL). LDL is the main carrier of cholesterol in the bloodstream. Both C and PS accumulate in the arterial wall and are associated with cardiovascular events

Cholesterol and phytosterols are absorbed in the small intestine (Fig. 1). They are solubilized and incorporated into micelles and absorbed via a specific carrier — Niemann-Pick C1-like protein 1 (NPC1L1) [12]. The heterodimer ATP binding cassette transporter G5 and G8 (ABCG5/8) secretes phytosterols and small amounts of cholesterol back into the intestinal lumen [13, 14]. The same processes occur in the hepatocyte, where both NPC1L1 and ABCG5/8 are expressed. As a result of this selection, which can be interpreted as a “defense mechanism” against phytosterols, plasma plant sterol concentration is 1000-fold lower than plasma cholesterol levels.

## Plant Sterols in Nutrition and Nutraceuticals

Phytosterols are part of plant foods, mainly in unrefined vegetable oils, grains, nuts, and olive oil. A typical western diet contains equal amounts (approximately 400 mg) of both plant sterols and cholesterol each day. Unlike cholesterol, phytosterols are not synthesized in humans, phytosterols have no function in human organism and diet is their only source.

Cytellin — the first plant sterol product — was introduced to the market in the late 50 s. Due to its low palatability and poor bioavailability, it was withdrawn in the 1985 [15]. In the mid-1990s, a margarine enriched with phytosterols — the first phytosterol containing functional food — was launched. Phytosterol-enriched low-fat milk and in margarines resulted in a 10–15% LDL-C reduction [16], whereas fortified cereals reduce LDL-C by 5.4% [17]. Up to date, different food products enriched with phytosterols are available: milk, soy, yoghurt products; soy and fruit drinks; cereal; sausage etc. More than 300 million USD are spent annually on functional food enriched with phytosterols worldwide [18].

## Plant Sterols and Cardiovascular Risk

### In Vitro Studies

Phytosterol cellular uptake was studied in HepG2 and CaCo-2 cell lines. In both cell lines, incubation with phytosterols resulted in the reduction of cellular cholesterol levels [19, 20]. Cytotoxic effects of phytosterols on human endothelial cells have been reported by Boberg and colleagues. They incubated human endothelial vein cells (HUVEC) with sitosterol for 72 h, which resulted in contraction of HUVEC and increase of intracellular lactate dehydrogenase [21]. In another study, Bao and colleagues reported a sitosterol-induced necrotic death in macrophages, derived from mice [22]. The lesional necrosis of atherosclerotic plaque mainly consists of macrophages; therefore, the authors hypothesized that sitosterol-induced accelerated macrophage death leads to plaque necrosis, to plaque rupture, and eventually to cardiovascular events. They supported this hypothesis by findings from a previous study in which they demonstrated a decrease in lesional macrophages resulting in a decrease of plaque necrosis in vivo [23]*.* They also speculated that sitosterol possibly inhibits the caspase pathway in macrophages, since in their model, sitosterol induces necrosis, but not cell-programmed apoptosis [22].

### In Vivo Studies

Animal studies demonstrate that a diet supplementation with phytosterols reduces serum cholesterol levels and atherosclerotic lesions [24–27]. However, there are also studies that demonstrate negative vascular effects. In apoE^−/−^ mice on a western-type, we found that lipid-lowering with a plant sterol-enriched diet compared to ezetimibe treatment resulted in twice as much atherosclerotic lesion formation [28]. This result is of particular interest since both options of inhibition of cholesterol absorption resulted in comparable serum cholesterol levels of 400 mg/dl. Similar results were obtained in apoE^−/−^ mice on a regular diet without cholesterol supplementation. Mice which were on a plant sterol-enriched diet demonstrated twice as much atherosclerotic lesions, compared to those treated with ezetimibe, even though serum cholesterol levels in both groups were 200 mg/dl. Moreover, wild-type mice supplemented with phytosterol-enriched food had increased cerebral lesion size after cerebral ischemia and impaired endothelial-dependent vasorelaxation compared to mice on normal chow. Further, Chen et al. reported an elevation of blood pressure in rats fed with phytosterol-enriched food [29].

Plat and colleagues investigated oxidized phytosterols. They fed LDL-r^−/−^ mice with control diet, oxysterol-enriched, and oxyphytosterol-enriched diet. The latter resulted in significant increase of oxyphytosterol amounts in atherosclerotic lesions compared to control and oxysterol-enriched diet. The proportion of severe atherosclerotic lesions was significantly higher on an oxysterol- and oxyphytosterol-enriched diet [30]. They concluded that oxidized phytosterols may contribute to the severity of atherosclerotic lesions. Although, in normal diet, only very small amounts of oxidized phytosterol can be found, they are elevated in the plasma of patients with sitosterolemia and small amounts can even be found in the plasma of healthy individuals [31].

Yu and colleagues generated ABCG5/8 knockout mice to elucidate the role of these genes. They observed a ~ 30-fold increase in sitosterol concentration in these mice on chow diet [32]. When dietary cholesterol content was increased, plasma phytosterol levels fell by ~ 50% (both in wild-type and ABCG5/8 knockout mice). Besides, liver and plasma cholesterol levels were decreased by 50% on chow diet, containing only very small amounts of cholesterol. Further analysis of knockout mice by Yu et al. and McDaniel et al. demonstrated that these mice had a normal phenotype comparable with that of wild-type mice. When fed with a high-phytosterol diet, they failed to gain weight, developed hepatosplenomegaly, and died prematurely. On a regular diet, they accumulated phytosterols in different tissues and organs and developed complex cardiac lesions [32, 33].

### Human Studies

#### Case Reports

Abnormally high levels of phytosterols are found in patients with sitosterolemia. Sitosterolemia is a rare genetic disorder, caused by mutations in ABCG5 or ABCG8 located in chromosome 2 resulting in increased plasma phytosterol levels (not mainly sitosterol but also campesterol, stigmasterol, and avenisterols). In 1974, two sisters were diagnosed with this rare disease presenting with xanthomatas (a deposition of phytosterols and cholesterol in different organs, mainly in extensor areas) and elevated phytosterol plasma levels [34]. The causal genetic mutation on ABCG5/8 was discovered only several years later in 2001 [13]. The clinical phenotype of sitosterolemia is very heterogenic — from asymptomatic to fatal cases as consequence of premature coronary heart disease. These patients are often characterized by xanthomas, severe coronary heart disease, aortic stenosis, and early cardiovascular death. Some patients present with hematologic disorders, such as stomatocytic hemolysis and macrothrombocytopenia [35]. The youngest patient diagnosed with sitosterolemia — a 2-year-old girl — had elevated LDL-C levels and tuberous and intertriginous xanthomas [36]. In another case, a 5-year-old girl died due to severe coronary three-vessel disease [37]. The discovery of this rare disease has raised the interest in plant sterol physiology and its potential damaging effects on the human organism.

#### Case–Control and Retrospective Cohort Studies

A meta-analysis of randomized clinical trials on lipid-lowering efficacy of plant sterols demonstrated that plant sterols reduce LDL-C levels by 17%. However, there is a great inter-individual variability [38••] and some studies have not found any LDL-C lowering effect of plant sterols at all [39, 40].

In a case–control study in females with documented coronary artery disease vs. healthy controls, elevated ratios of phytosterols to cholesterol were associated with increased cardiovascular risk [41]. Similar results were observed in men in the PROCAM (Prospective Cardiovascular Münster) study [42]. Our group demonstrated that among patients undergoing aortic valve replacement, those with a positive history of cardiovascular disease had an increased campesterol-to-lathosterol ratio in plasma and aortic valve cusps [43]. Correlation of enhanced phytosterol levels/ratio to cholesterol with cardiovascular risk and coronary heart disease was also reported in patients without diabetes history [44] and established CVD [45]. Glueck et al. analyzed phytosterol levels in 595 hypercholesterolemic subjects and its relation to the incidence of coronary heart disease in those individuals and their first-degree relatives. They found a weak correlation between phytosterol and cholesterol plasma levels and a positive correlation between the phytosterol concentrations and personal or family history of coronary heart disease [46]. Sudhop et al. also demonstrated a correlation between sitosterol, campesterol, sitosterol/cholesterol ratio, and campesterol/cholesterol ratio in individuals admitted to hospital for elective coronary artery bypass grafting [47].

On the other hand, there are case–control studies that show no effect on cardiovascular risk or a negative correlation of plasma phytosterol levels and cardiovascular disease. The prospective EPIC-Norfolk population study did not find any significant difference between those with coronary heart disease and healthy controls [48]. Similarly, the CORA study could not find any association between phytosterols and coronary heart disease [49].

#### Cohort Studies and Randomized Clinical Trials

LURIC (Ludwigshafen Risk and Cardiovascular Health Study) — a prospective cohort study with a total of 3,316 participants — demonstrated that plasma phytosterol levels were predictors of all-cause and cardiovascular mortality [50]. Another cohort study — MONIKA/KORA — demonstrated that in healthy men 35–64 years of age, higher phytosterol levels correlated with occurrence of myocardial infarction during 10-year follow-up [51]. On the other hand, a 22-year follow-up study of 232 men at high cardiovascular risk demonstrated that higher plant sterol levels are correlated with lower long-term mortality [52]. Most importantly, in patients admitted for coronary angiography for suspected coronary artery disease, we found that 7α-hydroxycampesterol and their ratios to cholesterol were associated with cardiovascular events during a 5-year follow-up period [53•]. All the above-mentioned studies are prospective cohort studies. Unfortunately, to date, there is no prospective, placebo-controlled, randomized trial to address the impact of phytosterol diet supplementation on hard cardiovascular outcomes.

#### Meta-analysis of Case–Control, Cross-Sectional, and Cohort Studies

A meta-analysis based on data from 17 studies (4 case–control, 3 cohort, 5 cross-sectional, and 5 nested case–control) evaluated the effect of phytosterol plasma levels on cardiovascular risk. The authors of this meta-analysis concluded that plasma phytosterol levels are not associated with cardiovascular risk. However, the results of this meta-analysis are to be interpreted with caution. First, there are no standardized methods of phytosterol measurements, hence results from different laboratories may differ largely. Second, not all studies were appropriately adjusted to potential confounding variables. Finally, of 59 initially selected publications, only 17 were included in the meta-analysis — another 41 were excluded because no explicit phytosterol exposure levels were reported or they did not meet inclusion criteria of this meta-analysis [54].

#### Genetic Studies

Plasma sterol concentrations are regulated by ABCG5/8 and NPL1C1. NPL1C1, which is expressed on the apical surface of enterocytes, is responsible for the intestinal absorption of both cholesterol and plant sterols [55], whereas ABCG5/8 reduces dietary sterol concentration in the plasma by pumping plant sterols and “excess” cholesterol back into the intestinal lumen [13]. Teupser et al. identified three variants of ABCG8 and ABO blood group locus, associated with increased plasma phytosterol concentration in humans. Around 10% of the variability in serum phytosterol levels in the normal population could be explained by these three variants. Teupser and colleagues conducted a meta-analysis of 11 different studies comprising over 13,000 cases with coronary heart disease and controls, and showed a positive correlation between those three alleles, serum phytosterol levels, and higher probability of coronary heart disease [56]. As early as 2010, Teupser and colleagues came to the conclusion that genetic variants that increase plasma plant sterol levels are associated with CAD.

A genome-wide study including sequence data of a total of 91,002 participants who were of European, African, or South Asian ancestry identified 15 distinct NPC1L1 inactivating mutations. Approximately 1 of 650 persons was a heterozygous carrier for one of these mutations. Even though heterozygous carriers of NPC1L1 inactivating mutations had a mean LDL-C level that was only 12 mg/dl lower than that of non-carriers, carrier status was associated with a relative risk reduction for CAD of 53%. The authors concluded that the genetic inhibition of NPC1L1 may also lower the risk of coronary heart disease by reducing the absorption of plant sterols [57].

Only recently, Helgadottir and colleagues [58••] reported a large genetic study from Iceland, Denmark, and the UK, with 85,544 cases and 648,442 controls. They determined a genetic risk score (GRS) for ABCG5/8 and NPC1L1 — lipid genes which are involved in plant sterol metabolism — and lipid genes, which are not involved in the plant sterol metabolism — such as PCSK9, apoB, HMG-CoA reductase, and LDL receptor. They identified variants of ABCG5/8 and NPC1L1, associated with a significant impact on non-HDL cholesterol and phytosterol levels. A predicted 1 mmol/L increase in non-HDL cholesterol for ABCG5/8 and NPC1L1 variants resulted in twofold increase of cardiovascular risk, whereas a 1 mmol/L non-HDL cholesterol increase for genes that affect only non-HDL cholesterol, but have no impact on plant sterol metabolism, such as PCSK9, apoB, HMG-CoA reductase, and LDL receptor, was associated with a 1.5-fold increase of cardiovascular risk. The authors concluded that non-HDL cholesterol can only explain around 60% of the increase in cardiovascular risk and the remaining 40% must be due to other mechanisms. ABCG5/8 and NPC1L1 are not associated with other conventional cardiovascular risk factors. In contrast, ABCG5/8 and NPC1L1 variants have a consistent close relationship to plant sterols — making elevated plant sterol levels a plausible explanation for the excess CAD risk. The authors, therefore, concluded that dietary sterols such as plant sterols may contribute directly to atherogenesis, raising questions about the safety of supplementing food with phytosterols for the purpose of cardiovascular risk reduction.

## Safety Concerns Beyond the Cardiovascular System

Besides proatherogenic effects, there are other safety concerns regarding phytosterols. As early as 2000, Ratnayake and colleagues found that vegetable oil rich in phytosterols makes red blood cells more rigid and less flexible and results in significant shortening of the lifespan of rats [59]. This finding leads to the ban of any phytosterol-enriched foods in Canada in 2003 [60].

Another concern coming from in vivo studies is the negative impact of phytosterols on hormonal status and the reproductive system of male and female rats [61, 62] and goldfish [63]. However, this finding could not be proven in humans. In 185 healthy volunteers, phytosterol consumption (1.6 g/day) for 1 year did not affect the reproductive hormone levels in both male and female participants [64].

Further, there is concern that phytosterols can interfere with the absorption of several fat-soluble vitamins — such as tocoferol and β-carotene [64, 65]. The Scientific Committee on Food of the European Commission has significant safety issues and recommends the use of natural sources of β-carotene to compensate for the reduction of β-carotenes caused by long-term consumption of phytosterol-enriched foods [66].

## Conclusions

The revised ESC/EAS guidelines for the management of dyslipidemias included for the first time a recommendation for plant sterols as part of lifestyle changes to reduce serum cholesterol levels. Recent genetic evidence suggests that plant sterols “per se” are atherogenic. These findings support the call for randomized controlled trials with hard cardiovascular outcomes prior to a general recommendation for plant sterols to lower serum cholesterol levels, as reiterated only recently by the German Cardiac Society (DGK).
